# Food insecurity is associated with self-reported oral health in school-age Ecuadorian children and is mediated by dietary and non-dietary factors

**DOI:** 10.1017/S1368980022002166

**Published:** 2022-09-29

**Authors:** M Margaret Weigel, Rodrigo X Armijos

**Affiliations:** 1 Department of Environmental & Occupational Health, Indiana University, School of Public Health, 1025 E. 7th Street, Bloomington, IN 47403, USA; 2 Global Environmental Health Research Laboratory, School of Public Health-Bloomington, Bloomington, IN, USA; 3 Center for Latin American & Caribbean Studies, Indiana University, Bloomington, IN, USA; 4 IU Center for Global Health, Indianapolis, IN, USA

**Keywords:** Child oral health, Food insecurity, Water intake, Fermentable carbohydrate foods, Toothbrushing frequency

## Abstract

**Objective:**

The main objective was to investigate the association of household food insecurity (HFI) with child oral health. A secondary objective was to explore potential dietary and non-dietary mediators of the HFI–child oral health relationship.

**Design:**

Cross-sectional data from the nationally representative Ecuadorian National Health and Nutrition Survey (2018) were analysed. The data included self-reported child oral health, HFI (Food Insecurity Experience Scale), diet (FFQ) and oral care behaviours (toothbrushing frequency, toothpaste use). The association of HFI with the reported number of oral health problems was examined with stereotype logistic regression. Parallel mediation analysis was used to explore potential dietary (highly fermentable carbohydrate foods, plain water) and non-dietary (toothbrushing) mediators of the HFI–oral health relationship. Bias-corrected standard errors and 95 % CI were obtained using non-parametric bootstrapping (10 000 repetitions). Effect size was measured by percent mediation (P_M_).

**Setting:**

Ecuador.

**Participants:**

5–17-year-old children (*n* 23 261).

**Results:**

HFI affected 23 % of child households. 38·5 % of children have at least one oral health problem. HFI was associated with a greater number of oral health problems: 1–2 problems (adjusted odds ratio (AOR) = 1·37; 95 % CI (1·15, 1·58); *P* = 0·0001), 3–4 problems (AOR = 2·21; 95 % CI (1·98, 2·44); *P* = 0·0001), 5–6 problems (AOR = 2·57; 95 % CI (2·27, 2·88); *P* = 0·0001). The HFI–oral health relationship was partially mediated by highly fermentable carbohydrate foods (P_M_ = 4·3 %), plain water (P_M_ = 1·8 %) and toothbrushing frequency (P_M_ = 3·3 %).

**Conclusions:**

HFI was associated with poorer child oral health. The HFI–oral health relationship was partially mediated by dietary and non-dietary factors. Longitudinal studies are needed to replicate our findings and investigate the role of other potential mediators.

Oral health is an essential component of overall child health and well-being^(1,2)^. The World Dental Federation defines oral health as the ability to speak, smile, smell, taste, touch, chew, swallow and emote without pain, discomfort and the presence of craniofacial disease^(3)^. Oral health exists along a continuum and is influenced by individual experiences, perceptions, values and other characteristics^(3,4)^. An estimated 573 million children worldwide suffer from poor oral health including caries and periodontal disease, the two most common oral conditions affecting this age group^(5)^. Poor childhood oral health has been linked to increased school absenteeism, poorer academic performance, lower quality of life and other adverse outcomes that often track into adulthood^(6,7)^.

Household food insecurity (HFI), the limited or uncertain availability of households to obtain nutritionally adequate and safe foods, is a well-documented, poverty-linked social determinant of health affecting an estimated 41 % of children aged < 15 years worldwide^(8)^. HFI is associated with adverse child mental and physical health outcomes^(9)^. However, the relationship of HFI with oral health is understudied in school-age children especially those from low-and-middle income countries (LMIC). Except for Brazil^(10–12)^, published studies are limited to three high-income countries, i.e. the USA^(13–16)^, New Zealand^(17,18)^ and South Korea^(19)^. This is an important gap in knowledge since both the population burdens of HFI^(20)^ and childhood oral disease are elevated in Latin American and other LMIC compared to high-income countries^(5,21)^. In addition, countries in the same geographical regions frequently differ from their other LMIC neighbours in their access to safe, nutritious food and dietary intake patterns, access to dental insurance and preventative oral care and treatment.

Diet, especially high carbohydrate intake, has been frequently identified in the literature as a potential mediator of the relationship between HFI and poor oral health. Food insecure households may be more likely to purchase and consume processed carbohydrate foods because of their cheaper cost and/or use of these to self-medicate or binge eat^(22)^. The frequent consumption of cheaper highly fermentable carbohydrate foods (e.g. processed snacks, fast foods, sugar-sweetened beverages) has been hypothesised as playing an important potential role in the development of oral disease in children from food insecure households^(13,15,23)^. This is a plausible hypothesis since such foods are reported to foster oral environments favourable to the development of caries, gingivitis and other periodontal disease. Specifically, the frequent consumption of highly fermentable carbohydrates provides acid-producing bacteria present in the oral cavity with a supply of free sugar substrates allowing for the formation of biofilms that cause tooth decay and inflammation, promoting periodontitis^(24)^. In addition, many soft drinks and other carbonated beverages contain high levels of phosphoric, carbonic and/or citric acid which can also erode tooth enamel and provide oral environments favouring bacterial growth and biofilm formation^(25,26)^.

Plain water consumption is a potential, unexplored dietary factor that could play a role in the HFI–child oral health relationship. The frequent consumption of plain water is documented to provide multiple oral benefits. These include washing away food residues, diluting bacterial acids, helping to re-mineralise tooth enamel and preventing dry mouth, a known risk factor for caries and periodontal disease^(27)^.

Toothbrushing is an important modifiable risk factor for childhood caries. Food insecurity has been linked with less frequent tooth brushing in children^(12,28)^. Toothbrushing behaviour is a plausible non-dietary pathway that may influence the HFI–child oral health relationship.

For example, children living in food insecure homes, especially those more severely affected, may eat fewer meals/d or skip eating which may cause them to brush less frequently or influence their parents to remind them to do so. Since food insecurity has been linked to increased stress, anxiety, depressive symptoms and psychosocial dysfunction in both children and mothers^(29)^, this could cause children to be less likely to perform self-care including regular toothbrushing and also reduce maternal caregiver ability to provide adequate care including regular tooth brushing.

As noted previously, the important question of whether HFI is associated with poorer oral health in school-aged children has not been examined in Latin American populations except for Brazil^(10–12)^. Ecuador is a middle-income Latin American country with a high documented population prevalence of both HFI^(20)^ and untreated childhood caries and periodontal disease^(30)^. We analysed data from a large, nationally representative Ecuadorian health and nutrition survey to investigate our primary research question of whether HFI is associated with poorer oral health in school-age children. Our secondary research question was to examine potential mediators of the hypothesised HFI–child oral health relationship.

Our primary hypothesis was that HFI would be associated with poorer child oral health as indicated by the number of self-reported oral health-related problems. We also hypothesised that two dietary factors (i.e. highly fermentable carbohydrate foods, plain drinking water) and oral hygiene behaviour (i.e. toothbrushing frequency) would be mediators of the HFI–oral health relationship. Specifically, we posited that the more consumption of highly fermentable foods would be associated with poorer self-reported child oral health while the greater consumption of plain water and more frequent toothbrushing would be associated with better child oral health.

## Methods

### Study design and population

We analysed cross-sectional data from the 2018 Nutrition and Health National Survey (Spanish acronym: ENSANUT), a national survey conducted every 5 years by the Ecuadorian National Institute of Statistics and Census^(31)^. The survey design, sampling method and other methodological characteristics have been previously described in detail^(31)^. Briefly, the probabilistic survey used a two-stage stratified sampling design to collect data from a nationally representative sample of persons living in 46 638 households from twenty-four provinces. The sampling frame for the survey was drawn from the Ecuadorian population census (2010–2017). The research universe consisted of all households and non-institutionalised persons from those households^(31)^. Eighty percent of the survey data were collected from November 2018 to January 2019 and the last 20 %, during June – July 2019^(31)^. Thus, all data collection occurred prior to first confirmed case of COVID-19 in Ecuador (February 29, 2020).

The database underwent a systematic evaluation by INEC personnel to identify any missing or improbable values^(31)^. Data were double-checked for discrepancies against that recorded in the written interview documents by field interviewers. If the written instrument was found to contain missing or improbable values, then the respondent was contacted by telephone to obtain and/or verify the information. In cases where it was not possible to contact the respondent by phone, a survey team member returned to the respondent’s household to obtain the information. Using this process, fewer than 1·2 % of the responses in the larger database were unable to be corrected or verified. In these few cases, where this occurred, missing or improbable data were imputed into the database by survey statisticians using conditional measures methodology^(31)^.

Our study analysis used the de-identified data from two ENSANUT 2018 survey modules. The Risk Factors Module for Children 5–17 years included self-reported data on child oral health, diet, dental visits and oral hygiene behaviours collected during in-person interviews with the parents of child participants aged 5–10 years and from children aged 11–17 years old^(31)^. To prevent oversampling, the survey selected only one 5–17-year-old child from each household to participate in the Risk Factors Module. The number of cases available for analysis was 23 261. The Household Module data were collected from household heads during in-person interviews^(31)^. We linked this to the Risk Factors Module in order to obtain additional data on participant and household socio-demographic characteristics and household food security status.

### Measures

#### Household food insecurity

HFI was the primary exposure of interest in our study. It was measured in the survey using the Food Insecurity Experience Scale (FIES), an experience-based instrument, administered to household heads^(32)^. The FIES includes eight questions (yes/no) that are focused on the self-reported, food-related behaviours and experiences of households associated with difficulties in accessing food due to lack of money or other resource constraints during the past 12 months^(32)^. Affirmative responses to the eight items were summed to produce a score for each household. These ranged from 0 to 8 with higher scores indicating more severe HFI.

We used the FIES online app to conduct a statistical validation of the food security data^(33)^. The FIES uses a probabilistic approach that allows for the classification of households according to their food security status. It also allows for the calculation of the prevalence of food insecurity that is comparable across populations. The app uses the Rasch model, a type of Item Response Theory. The Rasch model is widely used in health, education and psychology to measure unobservable traits through the analysis of responses to surveys and tests in this case, food insecurity experiences^(32)^. Briefly, the infit statistics for the eight individual items ranged from 0·86 to 1·17 indicating an adequate fit to the Rasch model. Residual correlations were < 4 for all item pairs. Rasch reliability for the 8-item scale was 0·75, indicating adequate reliability. In this study, households with 0–3 affirmative responses were classified as food secure and those with 4–8 with food insecurity.

#### Child oral health

The ENSANUT 2018 collected data on oral health problems reported by the child participants and their parents as indicated by mouth discomfort and pain, diminished mouth functioning and appearance and other manifestations. The child participants and/or their parents were asked whether the child had experienced certain problems caused by discomfort in the teeth or mouth during the past 12 months. These included pain, difficulties in chewing food, problems in speaking/pronouncing words, being unable or too embarrassed to smile, sleep disturbances or being unable to attend school. Responses were recorded as yes or no in the survey database. We summed the number of affirmative responses which ranged from zero (absence of problems) to six problems. These were collapsed into four categories for the analysis: 0 problems, 1–2 problems, 3–4 problems and 5–6 problems with higher scores indicating poorer oral health.

#### Dietary intake

Data collected from parents and children using an abbreviated FFQ were used to assess child intakes of fermentable carbohydrate foods for the past 7 d.

These included the number of d/week that child participants consumed any processed snack foods (e.g. crackers, cookies, potato chips, corn snacks, chocolates, etc.), fast foods from restaurants or street vendors (e.g. French fries, hamburgers, tacos, pizza, hot dogs, *salchipapas* (hot dog slices with fried potatoes)) or sweetened beverages (e.g. soft drinks, processed fruit juices, energy drinks). Data were also collected on the daily intake of plain water without any sweeteners, flavourings or colouring agents (number of glasses/d).

#### Oral health behaviour

Data on oral health behaviour collected from children/parents included recent dental care (any dental care during the past 12 months *v*. none). However, no information was provided by the survey as the specific reason for the child’s visit so it could not be ascertained whether this was for the purpose of a routine check-up, prophylaxis (e.g. topical fluoride application) or treatment for existing disease. The survey also collected data on usual toothbrushing frequency (0 times/week, 1 time/week, 2–3 times/week, 7 times/week, > 14 times/week) and use of toothpaste during teeth brushing (yes *v*. no). The survey did not collect data on whether the latter contained prophylactic fluoride.

#### Socio-demographic characteristics

Data collected on the socio-demographic characteristics of the sample included child age (years), gender (female *v*. male), self-identified ethnicity (mestizo ethnic majority *v*. ethnic minority), low maternal education (primary school or less *v*. secondary school and greater), urbanicity (rural *v*. urban) and geographical region of residence (Andean highlands, Pacific coast, Ecuadorian Amazon, Galapagos Islands). A proxy variable for material poverty (major household resources) was constructed from the individual asset variables identified in the survey. Households were classified as low resource (yes/no) if they did not own any of the following major assets: home, car or other motor vehicle, or major home appliance (refrigerator, freezer, washing machine, television, computer, etc.).

#### Data analysis

Statistical analyses were performed using STATA (*Stata Statistical Software: Release 17,* StataCorp., LLC, 2019) for the main research question in this study. Sample weights were applied to the summary, bivariate and multivariable analyses to account for the complex survey design of the 2018 ENSANUT. Statistical tests were two-sided and significance was set at *P* < 0·05. Participant socio-demographic characteristics were compared by household food security status using *X* ^2^ or Students’ independent *t*-test.

We used stereotypic logistic regression to estimate the association of household food security and number of reported oral health-related problems (0 problems, 1–2 problems, 3–4 problems, 5–6 problems). The adjusted model covariates included child age, gender, ethnicity, maternal education, household material resources, urbanicity and geographical region of residence. The results are reported as unadjusted and adjusted OR with 95 % CI.

For our secondary research question on potential mediators of the HFI–oral health relationship in children, we used the PROCESS macro for SPSS (v. 4.0) path analysis modeling tool^(34)^. In a mediation analysis, the relationship of *X* with *Y* is decomposed into a direct link and an indirect link. The total effect of X on Y is partitioned into a combination of a direct effect of *X* on *Y* and an indirect effect of *X* on *Y* that is transmitted through mediating variables (*M*). We used parallel mediation analysis as this approach allows for a more complex assessment of relationships and processes compared to single mediation^(35)^.

The parallel mediation analysis carried out in this study involved *X*, the independent variable (HFI), *Y*, the dependent variable (no. of oral health-related problems) and three mediator variables (*M*_*1–3*_) hypothesised as transmitting the causal effect of *X* to *Y*. It examined whether HFI’s effect on child oral health disappears or is reduced after the addition of the hypothesised mediators in the model. We tested the potential mediating variables step-by-step with the PROCESS macro. We aggregated processed snack foods, fast foods and sweetened beverages into a single group (highly fermentable carbohydrate foods) for the analysis. Plain water was the other dietary variable assessed for its mediation effects. The non-dietary mediator was toothbrushing frequency. The model estimated the total-, direct- and indirect effects and the proportion of total effect (P_M_) mediated by each mediator. It adjusted for child age, sex, ethnicity, maternal education, household material resources, urbanicity and geographic region of residence. Bias-corrected standard errors and 95 % CI were obtained using non-parametric bootstrapping (10 000 repetitions). The effect size for the mediation analysis was measured using percent mediation (P_M_), interpreted as the percent of the total effect accounted for by the indirect effects.

## Results

Table 1 displays the characteristics of the 23 261 Ecuadorian children in the study. The children ranged in age from 5 to 17 years with an average age of 10·6 years, 51 % were male and 82 % belonged to the mestizo ethnic majority group. Fifty-one percent had mothers who had only a primary school education or less, slightly more than half lived in low-resource households and most were urbanites. The sample was more or less evenly divided among children living in the Andean highlands, Pacific coastal plain or Amazonian regions of the country. Only 5 % resided in the Galapagos Islands region. Twenty-three percent of child households in the survey reported experiencing food insecurity during the previous 12 months.

**Table 1:** Characteristics of the 5–17-year-old child sample compared by their household food security status (*n* 23 261)

Characteristics	Total sample (*n* 23 261)		Food secure (*n* 12 832)		Food insecure (*n* 5293)	
	Weighted % or mean	se	Weighted % or mean	se	Weighted % or mean	se
Child gender						
Male	51·1		77·8		22·2	
Female	48·9		76·9		23·1	
Child age (years)	10·6	0·03	10·6	0·04	10·6	0·06
Child ethnicity						
Mestizo (ethnic majority group)	82·1		81·0		19·0*	
Ethnic minority (Indigenous, Afro-descendant)	17·9		60·5		39·5	
Maternal education						
Primary school or less	51·4		70·6		29·4*	
Secondary school or greater	48·6		84·5		15·5	
Low household assets						
Yes	54·0		78·0		22·0**	
No	46·0		76·6		23·4	
Residence location						
Urban area	61·0		81·2		18·8*	
Rural area	39·0		71·2		28·8	
Region						
Andean highland region	29·9		80·9		19·1*	
Pacific coastal plain region	32·8		78·4		21·6	
Amazonian region	32·1		69·9		30·1	
Insular (Galapagos Islands) region	5·2		96·1		3·9	
Any oral health problems	38·5		36·4		45·6	
Any oral health care visits in past 12 months†	53·9		55·1		49·6*	
Brushes teeth > 2 times/d	75·5		77·5		68·6	
Uses toothpaste when brushing teeth‡	98·5		98·6		98·2	
Highly fermentable carbohydrate food intake frequency§	1·5	0·01	1·6	0·01	1·4	0·02*
Processed snack foods intake frequency‖	1·6	0·02	1·7	0·02	1·5	0·03*
Fast food intake frequency¶	2·0	0·02	2·1	0·02	1·8	0·03*
Sweetened beverages intake frequency††	1·0	0·01	1·0	0·01	0·9	0·02*
Plain water intake‡‡	3·8	0·02	4·0	0·02	2·6	0·04*

* *P* = 0·0001.

** *P* = 0·001.

† Survey did not provide information on reason(s) for the visit so it is uncertain whether it was for a routine check-up, dental cleaning or other prophylaxis (e.g. topical fluoride application), or treatment of caries, extractions or other existing disease.

‡ Survey did not collect data on whether toothpaste contained fluoride or not.

§ Days that child consumed any sweetened beverages, processed or fast foods during past week.

‖ Days child consumed any processed snack foods such as cookies, crackers, candies, chocolates, corn-based snacks, potato chips and other processed foods during past week.

¶ Days child consumed any fast foods such as French fries, hamburgers, tacos, hot dogs, pizza, French fries with pieces of hot dogs, sausages, other toppings) purchased from fast food establishments during the past week.

†† Days child consumed sweetened beverages such as soft drinks, energy drinks, or processed fruit juice during past week.

‡‡ Number of glasses/d child consumed plain water without any colorants, sweeteners or flavours during past week.

Table 1 also compares the socio-demographic characteristics associated with HFI. The weighted analysis results revealed that a higher proportion of children from food insecure compared to food secure households belonged to an ethnic minority group, had poorly educated mothers, had low household resources and lived in a rural area, especially in the Amazonian region of the country. Fewer children from food insecure than food secure homes reported receiving any oral health care during the past 12 months. However, since the survey did not provide any information on the specific reason for the visit, it is uncertain whether any reported visits were for routine check-ups, prophylaxis (e.g. topical fluoride application), or treatment for existing oral disease. Most children reported brushing their teeth > 2 times/d with a greater proportion of food secure *v*. food insecure individuals doing so. Although almost all children (98·5 %) reported using toothpaste to brush their teeth, the survey did not collect data on whether the toothpaste they used contained prophylactic fluoride. As the table also shows, children living in food insecure homes consumed highly fermentable carbohydrate foods less frequently and drank less plain water compared to their food secure counterparts.

**Fig. 1:**
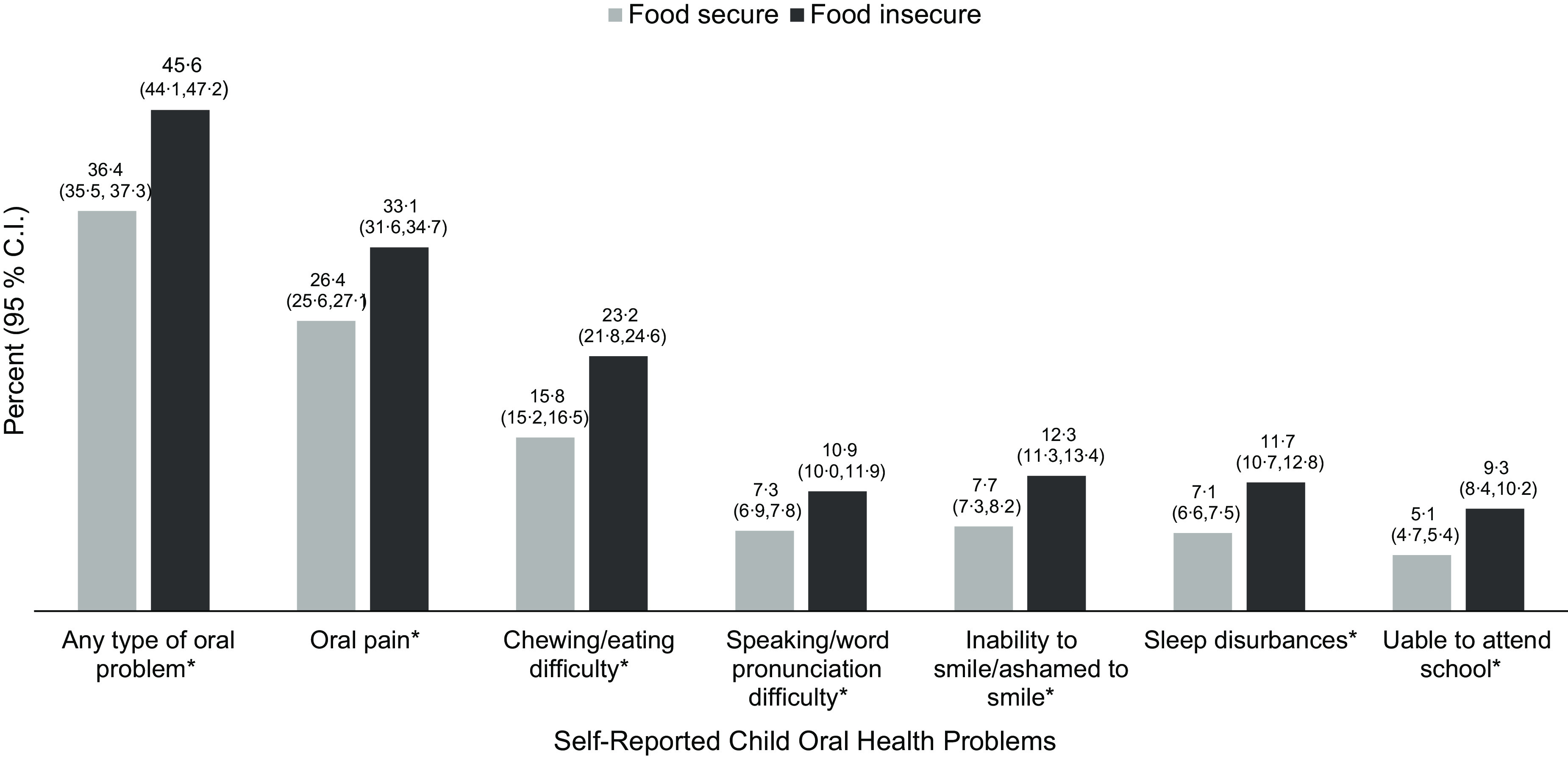
Proportion of 5–17-year-old children reporting any and specific oral health-related problems during past 12 months compared by household food security status^1^ (*n* 23 621). ^1^Contingency table analysis with *X* ^2^ (weighted sample). *P < 0·0001

Thirty-nine percent of children reported experiencing at least one oral health-related problems during the past 12 months. The most common of these was oral pain (27·9 %) followed by mechanical difficulties that made it difficult to chew/eat (17·5 %), smile (8·7 %) or speak/pronounce words (8·1 %) and sleep disturbances (8·1 %) and/or other oral cavity issues causing them to miss school (6 %). As Fig. 1 shows, a significantly larger proportion of children from food insecure compared to food secure households reported experiencing at least one oral health problem during the prior 12 months. Food insecure children also were more likely to report experiencing oral pain and other specific types of oral health problems.

As indicated in Table 2, 28·4 % of children reported experiencing 1–2 oral problems, 23·5 %, reported 3–4 problems and 22·7 % reported 5–6 problems in the past 12 months. The unadjusted stereotype logistic regression analysis results indicated that compared to the food secure children, those from food insecure households reported more oral health problems: 1–2 oral problems (OR = 1·77; 95 % CI (1·61, 1·94); *P* = 0·0001), 3–4 oral problems (OR = 3·11; 95 % CI (2·95, 3·27); *P* = 0·0001) and 5–6 problems (OR = 3·94; 95 % CI (3·78, 4·09); *P* = 0·0001). The inclusion of covariates in the adjusted model produced similar, although slightly attenuated, results: 1–2 problems (adjusted odds ratio (AOR) = 1·37; 95 % CI (1·15, 1·58); *P* = 0·0001), 3–4 problems (AOR = 2·21; 95 % CI (1·98, 2·44); *P* = 0·0001), 5–6 problems (AOR = 2·57; 95 % CI (2·27, 2·88); *P* = 0·0001).

**Table 2:** Stereotype logistic regression analysis of the association of household food security status with the number of self-reported oral health problems (*n* 23 261)

	Food secure (*n* 18 328)	Food insecure (*n* 5293)				
	Weighted %	Weighted %	Unadjusted OR	95 % CI†	Adjusted OR	95 % CI†,‡
Oral problem no.						
0 Problems	63·6	54·4*	Reference		Reference	
1–2 Problems	27·8	30·4*	1·77	1·61, 1·94*	1·37	1·15, 1·58*
3–4 Problems	7·3	12·3*	3·11	2·95, 3·27*	2·21	1·98, 2·44*
5–6 Problems	1·2	2·9*	3·94	3·78, 4·09*	2·57	2·27, 2·88*

* *P* = 0·0001.

† Model adjusted for child age (years), gender (male/female), ethnicity (ethnic minority/mestizo majority) low maternal education (any primary school or less/any secondary school or higher), major household resources (any/none), urbanicity (urban residence/rural residence) and geographical region of residence (Andean highlands, Pacific coast, Amazon, Galapagos Islands).

‡ Reported number of oral health problems in past 12 months (pain, difficulties in chewing food, problems in speaking/pronouncing words, being unable or too embarrassed to smile, sleep disturbances or being unable to attend school).

**Fig. 2:**
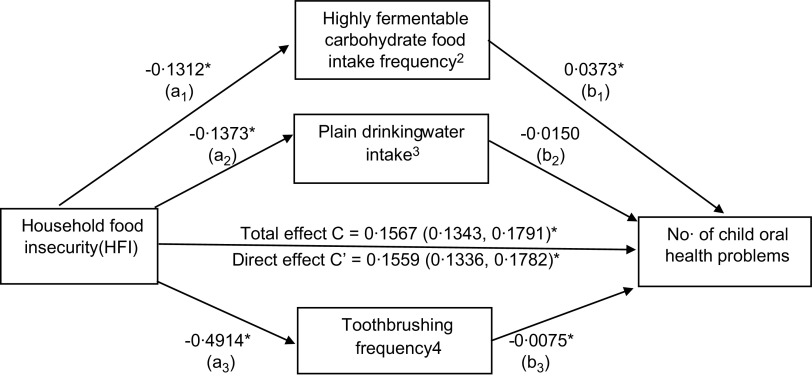
Process Macro path model diagram displaying findings from the adjusted parallel mediation model examining the contribution of dietary and non-dietary mediators in the HFI–child oral health relationship^1^. Highly fermentable carbohydrate food intake frequency indirect effects: *β* = -0·0049 (-0·0073, -0·0026)*; P_M_ = 4·3 %. Plain water intake indirect effects: *β* = 0·0021 (0·0010, 0.0033)*; P_M_ = 1·8 %. Toothbrushing frequency indirect effects: *β* = 0·0037 (0·0022, 0·0054)*; P_M_ = 3·3 %. *P = 0·0001. ^1^Model adjusted for child age (years), gender (male/female), ethnicity (mestizo, ethnic minority), low maternal education (any primary school or less/any secondary school or higher), any major household assets (any/none), urbanicity (urban residence/rural residence), region (Andean highlands, Pacific coast, Amazon, Galapagos Islands), tooth brushing frequency. ^2^No. of d/week child consumed one or more portions of processed snack foods, fast foods and sweetened beverages. ^3^No. of glasses/d child consumed plain water without any colorants, sweeteners, or flavours during past week. ^4^Usual toothbrushing frequency: 0 times/week, 1 time/week, 2–3 times/week, 7 times/week, > 14 times/week

Figure 2 shows the parallel mediation analysis findings which explored our secondary research question regarding the potential roles of dietary and non-dietary factors in mediating the HFI–oral health relationship in children. The total effect of HFI (c pathway) on the dependent variable (oral health problem number) was partially mediated by how frequently children consumed highly fermentable carbohydrate foods (a_1_b_1_ indirect pathway), plain water (a_2_b_2_ indirect pathway) and how frequently they brushed their teeth (a_3_b_3_ indirect pathway). Specifically, HFI was associated with lower intakes of highly fermentable foods (path a_1_) and plain water (path a_2_). It was also associated with lower tooth brushing frequency (path a_3_). The intake of highly fermentable carbohydrate foods was positively associated with the number of child oral health problems (path b_1_) suggestive of poorer oral health. Plain water intake (path b_2_) was negatively associated with the number of child oral health problems as was toothbrushing frequency (path b_3_).

The bootstrapping analysis identified a significant indirect effect of HFI on child oral health problems mediated through both dietary and non-dietary factors including highly fermentable carbohydrate foods (a_1_b_1_ = −0·0049; 95 % CI (-0·0073, -0·0026)), plain water (a_2_b_2_ = 0·0021; 95 % CI (0·0010, 0·0033)) and tooth brushing (a_3_b_3_ = 0·0037; 95 % CI (0·0022, 0·0054)) as their respective 95 % CI did not include zero. Proportion-mediated effect size estimates for the individual mediation pathways indicated that the two dietary mediators, highly fermentable carbohydrate foods and plain water, respectively, accounted for an estimated 4·3 % and 1·8 % of the total effect of HFI on the number of oral health problems. Toothbrushing frequency accounted for 3·3 %.

## Discussion

To the best of knowledge, this is the first study to examine the association of HFI with the oral health school-age children in a Latin American population other than Brazil. It is also the first to assess the potential role of HFI–oral relationship mediators in Latin American children. Consistent with our main hypothesis, the finding from this nationally representative study of 5–17-year-old children was that those living in food insecure households reported having significantly poorer oral health than their food secure counterparts. This finding is also consistent with prior studies reporting on similarly aged children from Brazil^(10–12)^ and several high-income countries^(13–19)^ despite differences in study designs, methods used to assess HFI and oral health and data analysis. The study also provided insights about potential dietary and non-dietary mediators of the HFI–child oral health relationship. These partial mediators together contributed a small (9·4 %) but significant proportion of the total effect of HFI on child oral health.

A poor diet, including the excess intake of cariogenic carbohydrate foods, has been suggested by prior authors as a potential mediator of the HFI–oral health relationship in children^(13,15,23)^. Our parallel mediation analysis results indicated that highly fermentable carbohydrate foods accounted for an estimated 4·3 % of the total effect of HFI on child oral health problems in the parallel mediation analysis. However, different from what we hypothesised, children from food insecure compared to food secure households consumed highly fermentable foods less rather than more frequently. The findings from the limited studies published on the association of HFI and the intake of highly fermentable carbohydrate foods in school-age Latin American children are mixed. Our study finding is consistent with what has been reported for school-age Venezuelan children^(36)^ but differs from studies conducted in Brazil^(37)^ and Mexico^(38)^ reporting higher intakes. The reason for the inconsistencies in study findings is unclear. In addition to differences in how HFI and diet were measured, they could also reflect differences in where these Latin American countries are in the nutritional transition as well as those related to national ‘junk food’ policies and regulations. These should be examined in detail by future studies.

Plain drinking water intake made a small but significant contribution to the HFI–oral health in our parallel mediation model. As far as we know, this is the first study to assess plain drinking water intake as a potential mediator of the HFI–child oral health relationship. In our study, food insecure children drank approximately around a third less plain water/d compared to those that were food secure and lower plain water intakes were associated with poorer child oral health. However, this did not appear to be the result of sweetened beverages displacing water as food insecure children also drank those less frequently. A more likely explanation is food insecure households were also water insecure. Our post hoc analysis of several WHO/UNICEF Joint Monitoring Program (JMP) household water indicators collected in the larger 2018 ENSANUT survey (*n* 42 071 households) indicated that a significantly greater proportion of food insecure compared to food secure households had drinking water that did not come from an improved source (23·8 % *v*. 11 %; *P* = 0·0001) and that was neither readily available (16·3 *v*. 7·3; *P* = 0·0001) nor safe (31·5 % *v*. 21·9 %; *P* = 0·0001). This concurs with emerging evidence from Latin American and other LMICs suggesting that food insecure households are also more likely to experience water insecurity due to intersecting cost and other access barriers^(39)^.

In addition to household water insecurity, future studies should also assess the potential mediating effects of natural and artificial fluoride sources in drinking water and food. Regional averages of natural fluoride present in Ecuadorian drinking water sources ranges from 0·06 to 0·31 ppm^(40)^, well below the optimal 0·7 ppm levels recommended to prevent tooth decay^(41)^. This is important since community water systems in Ecuador do not fluoridate drinking water. Instead, like many Latin American LMIC, supplemental fluoride is delivered to the population through a national salt fluoridation programme^(42)^. Thus, we surmise that the mediating effects of plain water consumption on the HFI–oral health relationship might be more pronounced among children living in those populations having community water fluoridation programmes.

Although inadequate toothbrushing behaviour is a well-documented modifiable risk factor for childhood caries, this is the first work to formally examine its potential as a HFI–child oral health relationship mediator. In our study, food insecure Ecuadorian children brushed their teeth much less frequently than those that were food secure, a finding is consistent with the only two studies to report on this issue, one in school-age Brazilian children^(12)^ and the other in U.S. preschoolers^(28)^. We surmise that the disrupted meals and other eating caused by HFI contributed to less frequent toothbrushing or alternatively, the adverse psychosocial state imposed by HFI reduced self-care by children and the caregiving ability of their parents and other family members to perform or oversee child tooth brushing results in poor oral health. Although the survey reported on toothbrushing frequency, it did not capture information on flossing behaviours or exposure to fluoridated toothpaste and fluoridated mouth rinses. These are associated with toothbrushing and could have an influence over and above its mechanical action on teeth and gums. Future studies should investigate toothbrushing behaviours and oral hygiene/care practices in more detail.

Some of the major strengths of this study include nationally representative survey data, a large sample size (*n* 23 621), a low non-response rate (1·19 %) and standardised data collection procedures and quality control methods^(31)^. Another strength was the use of a validated instrument (FIES) to measure HFI. One of the study limitations was the reliance on self-reports of oral health. Direct observation of caries in decidual or permanent teeth, gingivitis, missing teeth or other oral health conditions is preferrable but self- and parent-reported assessments are reported to be a valid and reliable proxy measures of oral health when clinical exam or dental records are not available^(43–46)^. Another is the potential for recall bias especially in younger individuals. Although recall bias may be more pronounced in younger children, in this survey, parents answered for those aged 5–10 years. Future studies should be undertaken to replicate our findings using objective clinical examinations to reduce this potential limitation.

It is possible that social approval/social desirability bias could have resulted from children/parents under- or over-reported child intake of foods and beverages perceived as more or less healthful or otherwise more or less socially desirable. The same could have been true of the responses of adult household heads regarding the food security situation of their households.

The dietary intake data collected in the 2018 ENSANUT consisted of only a few types of foods and the 7-d reference period differed from the 12-month reference period covered for HFI and oral health. However, this expected to have a limited effect on the association of diet with both HFI and oral health and the validity of our conclusions. Another limitation was that although the survey collected data on toothbrushing frequency and toothpaste use, it did not depend on other oral health behaviours that could have influenced child oral health such as dental floss or oral rinses, sealants and other fluoridated product use. The survey also did not collect data on child intake of fluoridated salt or measure fluoride levels naturally present in drinking water.

The study using 2018 ENSANUT survey data was cross-sectional. This type of design allows for inference but not the establishment of causal effects. This design may also have affected estimations of mediation for the dietary mediators examined in this study. A major criticism of mediation analysis performed on cross-sectional data is that of directionality, i.e. whether the temporal ordering of the independent and dependent variables is correct. Although possible, it seems to be unlikely that the poor oral health of children was responsible for food insecurity situation in their households. Dental care in Ecuador is constitutionally guaranteed, readily available and free through the public health system. However, in any case, because of the cross-sectional design, interpretations of our findings should be made with caution. Future studies should use longitudinal data to confirm the temporal order of the HFI–oral health food relationship and the dietary mediators identified in our study.

In conclusion, the study findings identified HFI as an important potential risk factor for poor oral health among school aged Ecuadorian children. Two dietary and one dietary mediators partially explained the association of HFI with poorer child oral health. Future studies should be conducted to replicate our study findings using longitudinal data as well as investigate the role of other potential mediators that may also mediate the HFI–child oral health relationship.
